# Dynamic encoding of phonetic categories in zebra finch auditory forebrain

**DOI:** 10.1038/s41598-023-37982-5

**Published:** 2023-07-10

**Authors:** Wanyi Liu, David S. Vicario

**Affiliations:** Department of Psychology, Rutgers, The State University of New Jersey, Piscataway, NJ 08854 USA

**Keywords:** Neuroscience, Psychology

## Abstract

Vocal communication requires the formation of acoustic categories to enable invariant representations of sounds despite superficial variations. Humans form acoustic categories for speech phonemes, enabling the listener to recognize words independent of speakers; animals can also discriminate speech phonemes. We investigated the neural mechanisms of this process using electrophysiological recordings from the zebra finch secondary auditory area, caudomedial nidopallium (NCM), during passive exposure to human speech stimuli consisting of two naturally spoken words produced by multiple speakers. Analysis of neural distance and decoding accuracy showed improvements in neural discrimination between word categories over the course of exposure, and this improved representation transferred to the same words by novel speakers. We conclude that NCM neurons formed generalized representations of word categories independent of speaker-specific variations that became more refined over the course of passive exposure. The discovery of this dynamic encoding process in NCM suggests a general processing mechanism for forming categorical representations of complex acoustic signals that humans share with other animals.

## Introduction

Perceptual invariance is the ability to recognize objects despite variations in the sensory inputs^1,2^. The detection of invariant features can subserve categorical learning. In a dynamic environment, the correct assignment of variable or noisy stimuli to appropriate functional categories is necessary for communication and individual recognition in both humans and other animals. The assignment of sounds to phonetic categories has been proposed to occur at different stages of language processing^3,4^, including auditory structures and dedicated language centers, but it remains unclear where and how the brain implements these categories.

Natural vocalizations inevitably include variations along dimensions that are not directly informative for communication. Both humans and other animals can identify recurring auditory signals consistently despite variations in acoustic features of individual exemplars. In human speech processing, one important example is the ability to recognize words regardless of variations across individual voices. This ability to form an invariant representation for phonetic categories is crucial for speech perception. Previous studies in non-human animals using both natural and synthetic human speech stimuli have suggested that the ability to discriminate human speech stimuli is not unique to humans^5–9^. Some studies also demonstrated that animals can form phonetic categories for human speech stimuli despite variations across individual speakers. For example, zebra finches trained in an operant conditioning paradigm can discriminate similar monosyllabic words produced by different human speakers^10^. When the stimuli were switched to the same words produced by novel speakers, zebra finches still performed the discrimination task at high accuracy, thus correctly applying the category rules to new exemplars. The successful learning of human words by non-human animals suggests that recognition of phonetic categories for human speech does not critically depend on human language abilities, but instead exploits general auditory pattern recognition functions that humans share with other species.

The neural mechanisms of invariant representation have been studied in the auditory systems of different animal species. In rats, population responses were more invariant to the distortions of ultrasonic vocalizations in the secondary auditory cortex than in the primary auditory cortex^11^. In songbirds, neurons in the secondary auditory area responded more similarly to calls belonging to the same vocalization type than in the primary auditory areas^12^. Neurons in the secondary auditory area of zebra finches can also form more invariant representations of song signals embedded in background noise than those in the primary auditory area^13,14^. These findings suggested that higher-order auditory areas may contribute to the invariant representation of auditory signals.

In the songbird auditory forebrain, the properties of the secondary auditory area, caudomedial nidopallium (NCM) suggest its potential role in the invariant representation of sound identity. Neural responses in songbird NCM are stronger for conspecific vocalizations than other sounds and exhibit a gradual stimulus-specific adaptation to repeated complex acoustic stimuli^15,16^. Selective adaptation to relevant stimulus features could provide a neural basis for encoding sound identity, because the defining properties of different sound exemplars of the same category are invariant and are thus heard more frequently, while other features vary. Critically, during the adaptation process, this decrease in evoked neural activity across stimulus repetitions can impact the response to some stimulus features more than others, reflecting a dynamic re-coding process that improves neural discrimination of auditory streams by adapting out invariant (and thus more frequent) features^17^. Thus the dynamic changes in encoding in NCM that reflect the frequency of exposure may improve discrimination and contribute to the formation of acoustic categories.

The present study investigated the effects of passive exposure to human speech sounds on auditory responses of NCM neurons in zebra finches. Multi-unit neural responses to two monosyllabic words spoken by ten speakers were recorded from the NCM of awake, restrained birds under passive listening conditions. Responses were compared between birds who had previously heard the same words spoken by ten other speakers and birds who heard the words for the first time. Analysis of neural responses showed that NCM neurons formed a generalized representation for word categories independent of individual variations that transferred to new exemplars, and this representation improved with passive exposure.

## Results

**Figure 1:**
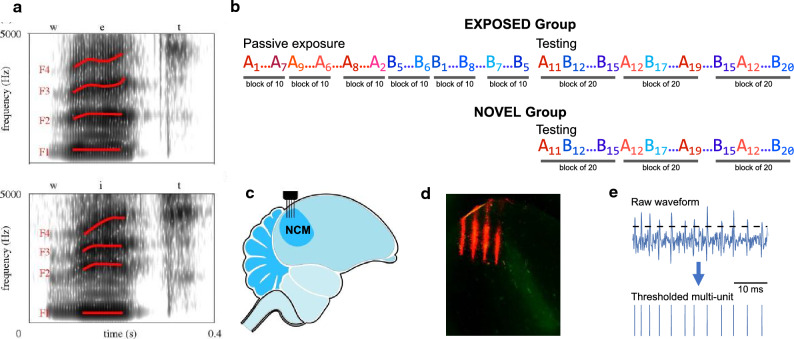
Acoustic stimuli, experiment design, electrophysiology, and histology. (**a**) Example spectrograms of the natural Dutch word stimuli. Top is a female voice saying *wet*, and bottom is a female voice saying *wit*. Used with permission of The Royal Society (U.K.), from Ohms et al.^10^; permission conveyed through Copyright Clearance Center, Inc. (**b**) Stimulus presentation orders and sessions (see "Methods" section). The EXPOSED group heard a passive exposure session in which two word stimuli (denoted as A and B; examples in 1a) produced by 10 human speakers (subscripts 1–10) were presented repeatedly. One word produced by the 10 speakers was presented in a pseudo-randomized order for 20 repetitions (20 blocks of 10 stimuli), followed by 20 repetitions of the other word produced by the same 10 speakers (20 blocks of 10 stimuli). In a subsequent testing session, the same two words produced by 10 novel human speakers were presented in a pseudo-randomized order in 10 consecutive blocks (each block consisted of 20 different stimuli). For the NOVEL group, the testing session was presented to the birds directly (no prior passive exposure session), using the same stimuli and presentation order as the testing session for the EXPOSED group. (**c**) Illustration of electrophysiological recordings. For each bird, two silicon probes were used to record the neural activity in NCM. Each probe was used for one hemisphere. (**d**) Histological verification of recording sites. The image shows one example of a sagittal brain section from one bird. Left is caudal white right is rostral. The bright green diagonal area indicates Field L. The four red lines show the trace of electrode shanks. (**e**) Illustration of the extraction of multi-unit activity from the raw recording waveforms by thresholding at 3 standard deviations from the mean).

To evaluate how passive exposure influences the neural representation of word categories, and whether the representation can be transferred to novel speakers, two groups of naïve adult zebra finches were exposed to two naturally spoken Dutch words (*“wit”* and *“wet”*, the same stimuli used by Ohms et al.^10^, Fig. 1a) under a passive listening condition. One group of birds (EXPOSED group: 8 male, 8 female) received an initial exposure to the words and the other group (NOVEL group: 11 male, 10 female) did not receive prior exposure. The EXPOSED group heard the two Dutch words produced by ten human speakers (prior exposure session), then was tested with the same two words produced by ten novel speakers (testing session). The NOVEL group was presented with the same testing session stimuli (Fig. 1b). Extracellular neural activity in NCM was recorded from awake, head-fixed zebra finches using electrode arrays (4-by-4 grid with 200 μm between sites) while birds were presented with the acoustic stimuli (Fig. 1c,d).

The influence of prior passive exposure on neural representation of word categories was evaluated by analyzing NCM responses to the same testing session stimuli (10 repetitions of the 20 different stimuli) in both the NOVEL and the EXPOSED group. Multi-unit spiking activity collected from 213 NCM sites in the EXPOSED group and 275 NCM sites in the NOVEL group was included in the subsequent analyses (Fig. 1e). Preliminary analyses did not show significant differences between hemispheres or sexes. Therefore, for all subsequent analyses, data from the two hemispheres as well as from male and female birds were combined. Neural responses recorded in the EXPOSED birds during the prior exposure session were analyzed separately (see Supplementary Figs. S1,S2).

**Figure 2:**
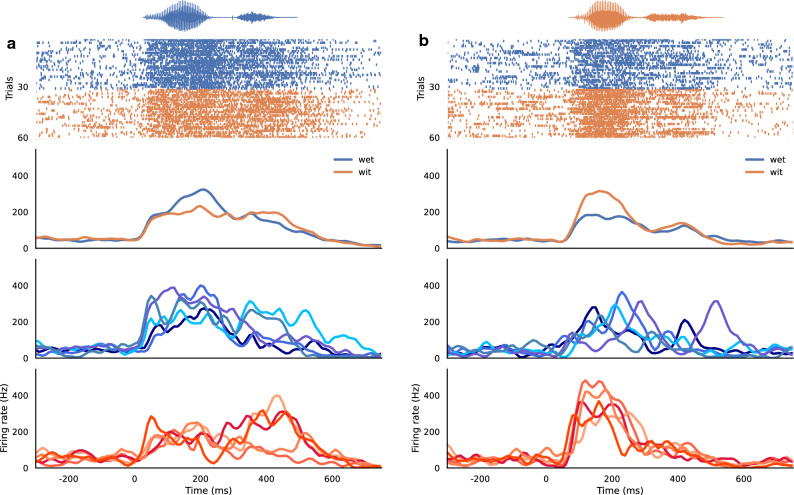
Representative multi-unit responses from two recording sites. (**a**) one site from the NOVEL group that has higher responses to the word *“wet”*. (**b**) one site from the EXPOSED group that has higher responses to the word *“wit”*. The top panel shows the amplitude envelope of the stimulus (an example for *“wet”* in (**a**) and *“wit”* in (**b**)) and the raster plot of spiking activity over 60 trials (30 for each word). The second panel shows the average peristimulus time histogram (PSTH) of the multi-unit activity for each word category. The bottom two panels show PSTHs for the responses to the words *“wet”* (blue colors) and *“wit”* (orange colors) as produced by the same 5 human speakers.

Examples of responses to the word stimuli from two representative multi-unit recording sites are shown as raster plots and as peristimulus time histograms (PSTHs) in Fig. 2. One site that showed stronger responses to the word *“wet”* (Fig. 2a) is from the NOVEL group, and another site that showed stronger responses to the word *“wit"* (Fig. 2b) is from the EXPOSED group.

### NCM responses adapt and show familiarity after prior passive exposure

**Figure 3:**
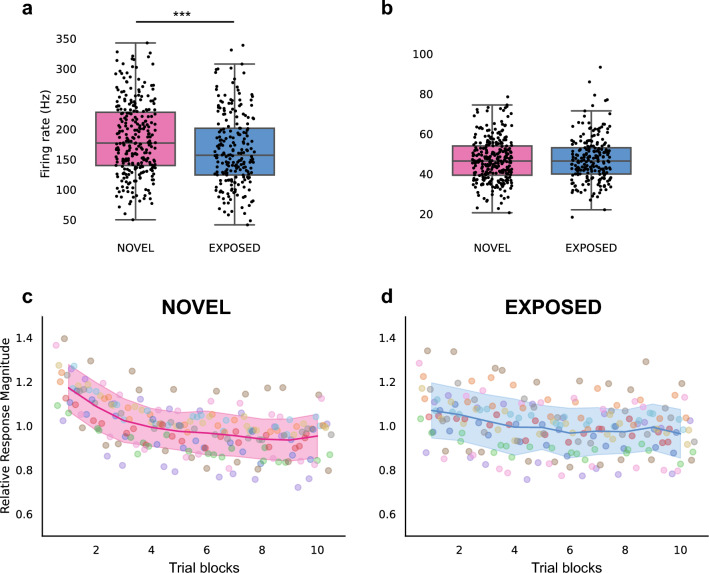
NCM firing rates and relative response magnitude (RRM) during the testing session. (**a**) Multi-unit firing rates during stimulus presentation for the NOVEL and EXPOSED groups. (**b**) Firing rates during the baseline period. (**c**) and (**d**) show the averaged RRM across the testing session. (**c**) The NOVEL group. (**d**) The EXPOSED group. Each dot represents the average RRM across all recording sites for each trial, and different colors indicate different stimuli. The average RRM across all 20 stimuli in one trial block (20 trials) are connected in colored lines, and the shaded areas represent the standard error of the RRM to all 20 trials in that block.

The average responses to word stimuli were compared between the NOVEL and EXPOSED groups. The multi-unit firing rates during the stimulus presentation (0–400 ms since stimulus onset) were significantly higher for the NOVEL group (Mean = 184.39 Hz, SD = 60.15 Hz) than for the EXPOSED group (Mean = 165.43 Hz, SD = 59.69 Hz. Mann–Whitney U test, *U* = 23,968, *p* < 0.001, Fig. 3a). The baseline firing rates (0–400 ms before stimulus onset) did not show any significant difference between the two groups (NOVEL: Mean = 47.02 Hz, SD = 10.77 Hz; EXPOSED: Mean = 47.37 Hz, SD = 11.15 Hz. Mann–Whitney U test, *U* = 29,566.5, *p* = 0.857, Fig. 3b).

**Figure 4:**
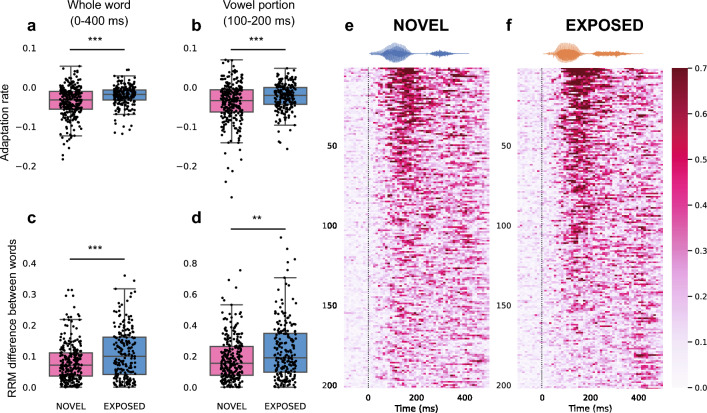
Adaptation rates and absolute difference in RRM between word categories in NCM for the NOVEL (pink) and EXPOSED (blue) groups. (**a**) The adaptation rates calculated with neural responses during the entire 400 ms (see "Methods" section). Each dot represents the adaptation rate from one multi-unit site. The box shows the quartiles of the dataset while the whiskers extend to the rest of the distribution, except for points that are determined to be outliers. (**b**) The adaptation rate calculated with neural responses during 100–200 ms since stimulus onset (the vowel portion). (**c**) The absolute difference in RRM between the two word categories during the 400 ms of stimulus presentation. (**d**) The absolute difference in RRM between the two word categories during 100–200 ms since stimulus onset (the vowel portion). **, *p* < 0.01. ***, *p* < 0.001. The absolute response difference between word categories across the time course of stimulus presentation was plotted for each site, for both the NOVEL group (**e**) and the EXPOSED group (**f**). Each row represents one multi-unit recording site. Only 200 sites from each group were shown. The color indicates the absolute response difference, which is calculated as the absolute value between the relative response magnitudes of the two word categories for 10-ms each time window for each site. A representative waveform of each word category is shown above to indicate the temporal structure of the stimuli in relation to the response difference below.

To account for the variability in response magnitudes between individual sites, the multi-unit firing rates were normalized for each site (relative response magnitude, RRM). When the average of RRM in NCM for all trials was compared across stimulus repetitions, responses in NCM showed a decrease in magnitude with repeated exposure (Fig. 3c,d). These changes were assessed with multilevel modeling and the rate of decrease was calculated as the adaptation rate for each multi-unit site using the RRM during stimulus presentation (0–400 ms since stimulus onset). For both groups, the adaptation rates were significantly lower than zero, indicating that neural responses decreased over stimulus presentation (NOVEL: *N* = 275, Mean = − 0.036, SD = 0.038, one sample Wilcoxon signed-rank test: *Z* = 2568, *p* < 0.001; EXPOSED: *N* = 213, Mean = − 0.021, SD = 0.025, one sample Wilcoxon signed-rank test: *Z* = 2103, *p* < 0.001). The adaptation rates were then compared between the NOVEL group and the EXPOSED group. We found that the adaptation rates were steeper for the NOVEL group than that of the EXPOSED group (Mann–Whitney U test, *U* = 36,573, *p* < 0.001, Fig. 4a). Similarly, the adaptation rate was also calculated using just the response during the vowel portion of the word stimuli (100–200 ms since stimulus onset). The adaptation rates were also significantly lower than zero for both groups (NOVEL: Mean = − 0.038, SD = 0.050, one sample Wilcoxon signed-rank test: *Z* = 4364, *p* < 0.001; EXPOSED: Mean = − 0.024, SD = 0.034, one sample Wilcoxon signed-rank test: *Z* = 3396, *p* < 0.001) and was also steeper for the NOVEL group (Mann–Whitney U test, *U* = 34,398, *p* < 0.001, Fig. 4b). This result suggested that after being passively exposed to the word stimuli, neural responses in NCM to the same words produced by novel speakers showed a slower adaptation process, consistent with prior results showing that the adaptation rate provides a measure of familiarity for acoustic stimuli^16,17^.

The absolute value of the difference in RRM to the word categories was calculated for both the entire stimulus duration and for the vowel portion, then compared between the two groups of birds (Fig. 4c,d). The absolute difference for each NCM site across the time course of stimulus presentation was shown in Fig. 4e,f. The absolute difference between word categories during the entire stimulus presentation period was higher for the EXPOSED group than that for the NOVEL group (NOVEL: Mean = 0.082, SD = 0.061; EXPOSED: Mean = 0.109, SD = 0.079. Mann–Whitney *U* test, U = 34,829, *p* < 0.001, Fig. 4c). The absolute response difference during the vowel portion was also higher for the EXPOSED group (NOVEL: Mean = 0.182, SD = 0.136; EXPOSED: Mean = 0.236, SD = 0.182. Mann–Whitney U test, *U* = 33,969, *p* = 0.002, Fig. 4d). This result indicates that passive exposure can lead to increased difference between word categories in neural response in NCM.

### Neural distance increases over repeated stimulus presentation

**Figure 5:**
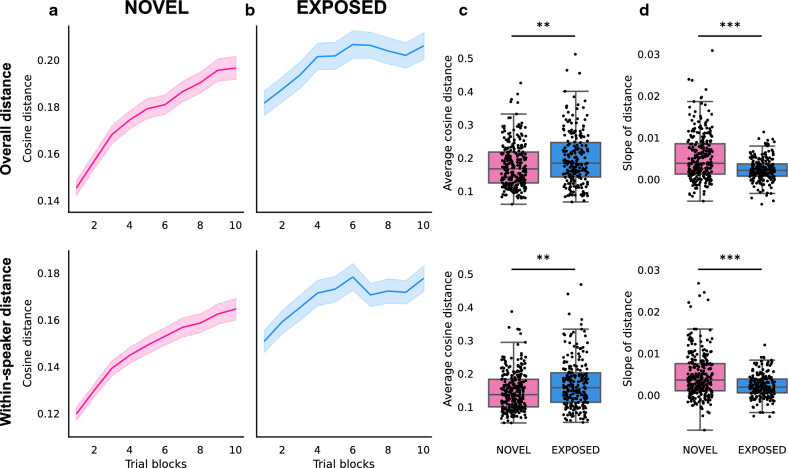
Neural distance over stimulus repetitions in the testing session. The cosine distance between neural responses to all stimuli (overall distance, top panel) and to the two-word pair produced by the same speaker (within-speaker distance, bottom panel) was calculated for each trial block (20 stimuli) for (**a**) The NOVEL group and (**b**) The EXPOSED group. Shaded areas represent the standard error of the neural distance across all the multi-unit sites. (**c**) The average cosine distance across all trial blocks was compared between the NOVEL group and the EXPOSED group for the overall distance (top) and within-speaker distance (bottom). (**d**) Regression slope was calculated to evaluate the change of cosine distance across trial blocks for the overall distance (top) and within-speaker distance (bottom). **, *p* < 0.01. ***, *p* < 0.001.

To further evaluate the changes in neural response patterns during passive exposure, the neural distance of response temporal profiles was calculated across stimulus repetitions for each multi-unit recording site (Fig. 5). Specifically, the overall neural distance was calculated as the averaged cosine distance (see "Methods" section and Supplementary Fig. S3) between all stimulus pairs for each block of 20 stimuli (“overall distance”, Fig. 5a,b, top), and the neural distance between words was calculated as the averaged cosine distance between each two-word pair produced by the same speaker (“within-speaker distance”, Fig. 5a,b, bottom). Both the overall distance and within-speaker distance were larger for the EXPOSED group (Mann–Whitney U test, overall distance: *U* = 33,806, *p* = 0.003; within-speaker distance: *U* = 33,971, *p* = 0.002, Fig. 5c). In addition, the slopes of linear regression models between neural distance and trial block numbers were calculated to evaluate the change in neural distance (Fig. 5d). The neural distance increased over stimulus repetitions in both NOVEL and EXPOSED groups (one-sample Wilcoxon tests, all *p-*values < 0.001). The rate of increase was higher for the NOVEL group (Mann–Whitney U test, overall distance: *U* = 20,382, *p* < 0.001; within-speaker distance: *U* = 21,462, *p* < 0.001). This result indicates that neural responses to the two word stimuli become more dissimilar through passive exposure.

### Population response to words changes over repeated stimulus presentation

**Figure 6:**
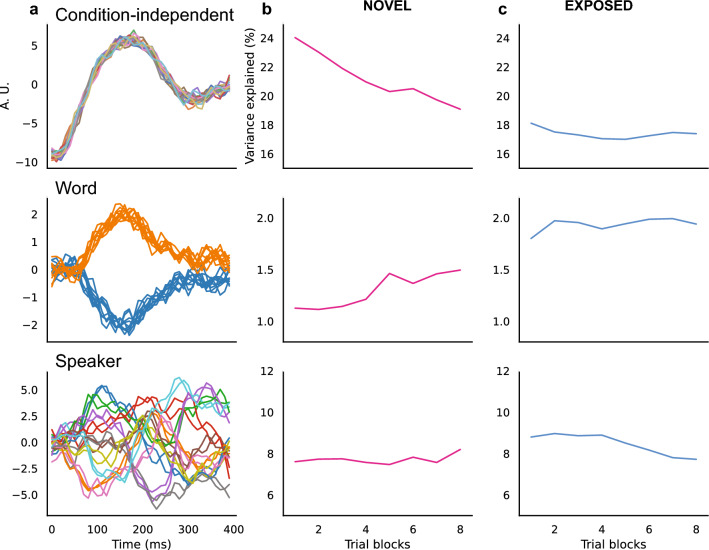
Demixed PCA components of NCM population response and the variance explained by each component. (**a**) Examples of the first condition-independent (time) component (top), the first word component (middle), and the first speaker component (bottom) for the NOVEL group. In each subplot, there are 20 lines that correspond to the response to the 20 stimuli. For the condition-independent component, each color corresponds to each individual stimulus. For the word component, each color corresponds to each word. For the speaker component, each color corresponds to each speaker. The X axis is the time since stimulus onset, and the Y axis is arbitrary units (A. U.) that are related to neural response magnitudes. (**b**) and (**c**) show the changes of variance explained by each component across trial blocks in the testing session for the NOVEL group (**b**) and the EXPOSED group (**c**) respectively.

To identify the representation of acoustic components by neuron populations, demixed principal component analysis (dPCA)^18^ was applied to decompose neural population activity into components that capture the variances and are interpretable with respect to the specific experiment parameters. Multi-unit activity was used to create a pseudo-population in this dimensionality reduction approach, as previous studies showed that low dimensional population dynamics can be extracted using multi-unit instead of single-unit activity^19^. As seen in Fig. 6a, the condition-independent component (top) showed a common response pattern across the 400 ms stimulus period for all conditions and captured most of the population variance. The word component (middle) captured the population variance for word categories, which divided into two functions that correspond to the two words. The speaker component (bottom) captured the variance explained by individual speakers and showed a unique pattern for each individual speaker.

To evaluate the dynamic change of variance explained by the three different components over stimulus repetitions, the same dPCA analysis was applied using trial blocks across stimulus repetitions, and the change of variance explained for each component over stimulus repetitions is shown in Fig. 6b,c. For the NOVEL group, the condition-independent component showed a decreasing trend over stimulus repetitions (Fig. 6b top); a linear regression showed that this variance decrease was significant (*t*(6) = − 9.774, *p* < 0.001). In addition, the variance explained by the condition-independent component (Fig. 6c top) was lower for the EXPOSED group (Mann–Whitney U test, *U* = 0.0, *p* < 0.001), but did not decrease significantly over stimulus repetitions (*t*(6) = − 1.259, *p* = 0.255,). This pattern suggests that the population representation becomes more dissimilar for different stimuli over repetitions.

The variance explained by the first word component showed an increasing trend over stimulus repetitions for the NOVEL group (Fig. 6b middle); a linear regression showed that this increase was a significant effect (*t*(6) = 5.852, *p* = 0.001). For the EXPOSED group, the variance explained by the first word component was higher than for the NOVEL group (Mann–Whitney U test, *U* = 0.0, *p* < 0.001), but did not increase significantly with stimulus repetitions (Fig. 6c middle, *t*(6) = 1.690, *p* = 0.142). This pattern suggests that passive exposure increased the variance explained by word categories.

The variance explained by the first speaker component (Fig. 6b bottom) did not show a clear increasing or decreasing trend for the NOVEL group (*t*(6) = 1.224, *p* = 0.267), but showed a small decrease over repetitions for the EXPOSED group (*t*(6) = 5.526, *p* = 0.001, Fig. 6c bottom). The variance explained by the first speaker component was higher for the EXPOSED group (Mann–Whitney U test, *U* = 7.0, *p* = 0.005).

### Population response forms a generalized representation of word categories

To illustrate that word identities can be represented in a generalized form, a decoding-based approach was applied to classify word identities using the responses of the NCM neuron population. The training and validation of the classification algorithm used neural responses to words produced by different sets of speakers. If the decoding algorithm can be generalized to classify words produced by novel speakers, then it indicates that the neural responses can form a generalized representation of word identity across individual variants.

**Figure 7:**
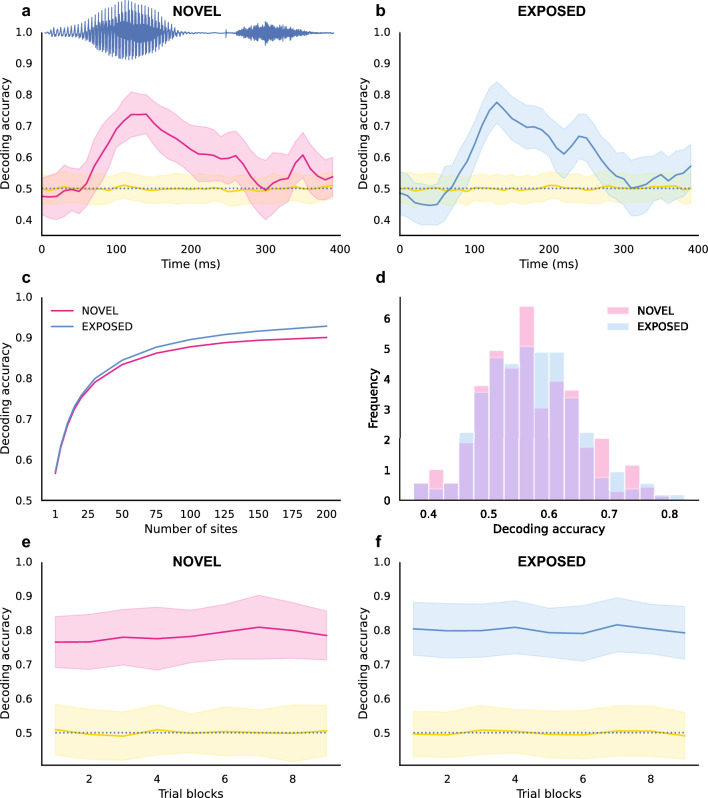
Temporal dynamics of the decoding accuracy for word categories, and the decoding accuracy across different sites and stimulus repetitions. The population decoding accuracy for word categories was calculated across the time since stimulus onset for (**a**) The NOVEL group. (**b**) The EXPOSED group. For each group, the decoding accuracy was calculated by randomly selecting 30 sites from all NCM sites for 100 iterations. The pink and blue lines represent the decoding accuracy calculated from the neural data, while yellow lines represent the shuffled control (see "Methods" section). The shaded areas represent the standard deviations across iterations. The decoding accuracy is the highest around the time when the vowel portion is presented (as is shown in the example waveform of one word stimulus). (**c**) Decoding accuracy increases as more recording sites are randomly selected in both NOVEL and EXPOSED groups. (**d**) The distribution of decoding accuracy for individual sites. The decoding accuracy was calculated across trial blocks in the testing session for (**e**) the NOVEL group and (**f**) the EXPOSED group.

We applied this decoding algorithm to each 10-ms time window during the stimulus presentation period (0–400 ms since the stimulus onset) to evaluate if the population responses can capture the acoustic differences between word categories. Figure 7a,b show examples of how the decoding accuracy changes over time. The decoding accuracy was relatively higher at around 100–200 ms after stimulus onset and slightly later for both the NOVEL and EXPOSED groups. This corresponds to the vowel portion that differs and is a distinguishing feature between the two word stimuli. This suggests that the decoding algorithm can capture neural responses that differentiate relevant acoustic features—in this case, the vowel segment that distinguishes “*wit*” and “*wet*” (cf. Figure 1a).

To investigate the contribution of individual sites to these population phenomena, the decoding accuracy for word categories was then calculated for different numbers of sites. Figure 7c shows the mean decoding accuracy as a function of the number of randomly selected sites (12 different numbers of sites, see "Methods" section). For both groups, decoding accuracy increased as more sites were included. Decoding accuracy was significantly higher for the EXPOSED group when the number of selected sites was equal to or larger than 30 (Mann–Whitney U tests with a Bonferroni adjusted alpha level of 0.00083, based on an uncorrected alpha level of 0.01, divided by the number of comparisons, in this case, 12). We also applied this decoding approach to individual sites to evaluate their contribution to population decoding (Fig. 7d). The mean decoding accuracy was slightly higher for the EXPOSED group (Mean = 0.571, SD = 0.077) than the NOVEL group (Mean = 0.568, SD = 0.080), but the difference was not significant (Mann–Whitney U test, *U* = 28,663, *p* = 0.687).

To evaluate how the decoding accuracy changed over stimulus repetitions, the decoding approach was applied using trial blocks across stimulus repetitions (Fig. 7e,f). As the responses during the vowel portion may include the actual difference in the stimuli for discrimination, only neural responses to the vowel portion (100–200 ms) were used. For each group, the decoding algorithm was applied to the population responses over stimulus repetitions using random selections of 30 recording sites from all NCM sites for 100 iterations. Results of linear regression models between averaged decoding accuracy and trial block numbers indicated that there was a small increase in decoding accuracy for the NOVEL group (*t*(7) = 3.388, *p* = 0.012), but no significant change for the EXPOSED group (*t*(7) = − 0.222, *p* = 0.831).

### Subgroups of NCM sites make different contributions

**Figure 8:**
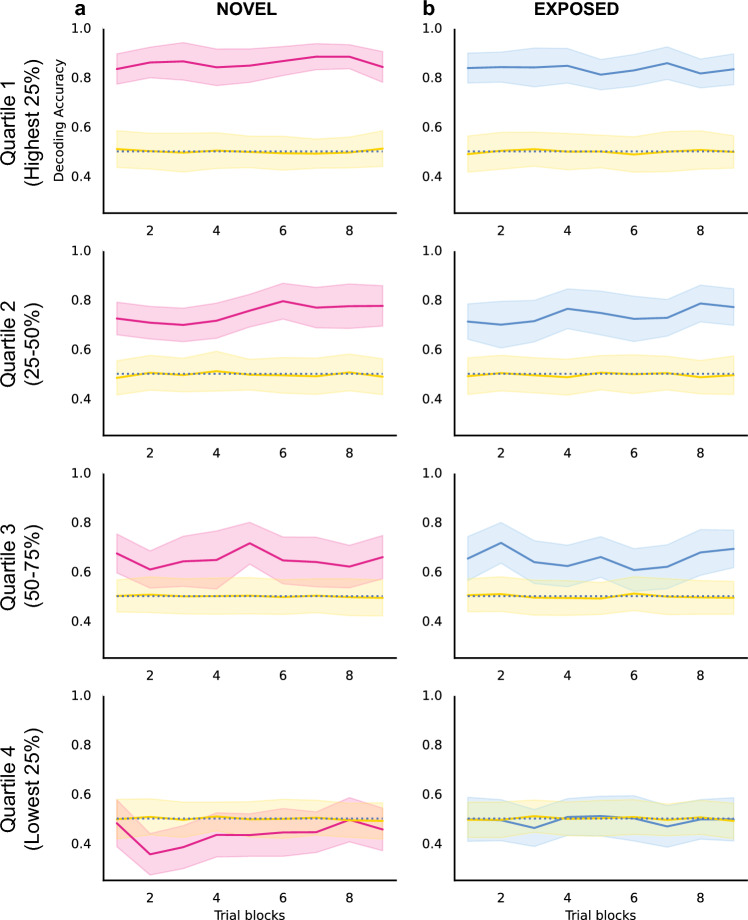
Decoding accuracy for subgroups of NCM sites. For both NOVEL (**a**) and EXPOSED (**b**) groups, all NCM recording sites were divided into 4 quartiles based on the decoding accuracy of individual sites. Each group consists of ~ 1/4 of all recording sites. Each row represents one subgroup, from the highest 25% (top) to the lowest 25% (bottom). Decoding accuracy was calculated in the same way as in Fig. 7, except that 20 randomly selected sites were used for each iteration. The pink and blue lines represent the decoding accuracy calculated from the neural data, while yellow lines represent the shuffled control (see "Methods" section). The shaded areas represent the standard deviations across iterations.

To evaluate the properties and contribution of individual NCM sites, we further divided all recording sites of each group into 4 quartiles based on their individual decoding accuracy, with one quarter of all sites in each subgroup. The decoding accuracy over stimulus presentation was evaluated for each quartile respectively (Fig. 8). For both NOVEL and EXPOSED groups, sites in the top 3 quartiles decoded word categories well above chance level across all repetitions (Wilcoxon signed-rank tests with a Bonferroni adjusted alpha level of 0.0011 (0.01/9)). In contrast, the quartile with the lowest performing sites did not show a significantly above-chance decoding accuracy across stimulus repetitions (Wilcoxon signed-rank tests with a Bonferroni adjusted alpha level of 0.0011 (0.01/9)).

The change of decoding accuracy across stimulus repetitions was estimated for each quartile with linear regression models, similarly to the approach for all NCM sites. For both NOVEL and EXPOSED groups, the second quartile (sites with decoding accuracy in the top 25 to 50%) showed an increase in decoding accuracy across stimulus repetitions (NOVEL subgroup: *t*(7) = 3.712, *p* = 0.008; EXPOSED subgroup: *t*(7) = 2.838, *p* = 0.025). For all other subgroups, the decoding accuracy did not show a significant increase or decrease across stimulus repetitions (all *p*s > 0.05). This suggests that the increase in decoding accuracy across stimulus repetitions may be primarily driven by a subgroup of sites. Sites with higher or lower decoding accuracy did not show significant changes over stimulus repetitions.

## Discussion

We investigated the effect of passive exposure on the neural representation of phonetic categories in zebra finch NCM using two naturally spoken words produced by different human speakers. Neural responses in NCM adapt over word repetitions, despite the considerable acoustic variations among individual human speakers. Moreover, the variance explained by word categories increases over stimulus repetitions as response magnitude decreases. Together, the findings support a dynamic change in population coding that improves the representation of word categories and becomes stronger after passive exposure. Decoding-based approaches showed that word identity can be predicted from neural responses to words produced by different human speakers. We conclude that NCM neurons can form representations for the word categories that generalize across individual acoustic variations.

The results show that neurons in zebra finch auditory forebrain can represent invariant features of acoustic stimuli. Using a population decoding approach, we showed that word identity can be predicted by training and validating the decoding algorithm with neural responses to words produced by different human speakers. This supports the idea that NCM responses can form a generalized representation for word categories despite individual variations. However, as individual sites showed differences in response pattern and decoding accuracy, we hypothesized that sites may also contribute differently to the discrimination between phonetic categories. A small subset of sites can show the same level of decoding accuracy as the entire population. This may indicate that a subset of the NCM neuron population has a pre-existing ability to differentiate phonetic categories. In contrast, nearly a quarter of NCM sites did not show any discrimination between word categories (they were at or below chance level) even after passive exposure. This may be due to the large variations in responses to different individual voices and selectivity for individual voice features rather than phonetic categories.

The current results we obtained in a higher auditory area of the songbird brain contribute to our general understanding of the neural mechanisms of speaker-independent perception of human speech sounds. We show that neural representations of phonetic categories in songbird NCM can be generalized across the variations among natural human voices. This speaker-independent representation can subserve the speaker-independent perception of phonetic categories seen in behavioral studies (e.g., Ohms et al.^10^) and is consistent with other results showing that many animal species can categorize human speech stimuli in behavioral tasks regardless of variation across individual voices^5–9^. Thus, this ability is not unique to humans, but may be based on fundamental processing mechanisms that humans share with other animals. Indeed, speaker-independent categorization in zebra finches can be generalized to novel speakers^10^ and can be achieved without prior exposure to speaker-related variability in speech sounds^20^. While previous physiological studies have demonstrated that categorical representation of human speech phonemes can be formed in the primary auditory cortex of non-human animals^21–23^, we provide evidence for this ability in a higher order area, where neural representations are more invariant to sound distortions or background noise than in the primary auditory area^11,13,14^.

The neural representation of phonetic categories changes dynamically with passive exposure and transfers to novel voices. Consistent with the existing finding that neural responses in NCM adapt to the repetition of acoustic stimuli^15^, we found that neural activity in NCM showed a decreasing response to the repetition of word stimuli (Fig. 3); this demonstrates the phenomenon of adaptation to word stimuli presented in an intermixed order. More importantly, in NCM the response adapts faster when the sound is novel (a steeper slope) than when the sound is familiar (a less steep slope). Therefore, a slower adaptation rate suggests the stimuli are more familiar. In our study, the adaptation rate is slower for the EXPOSED group than for the NOVEL group, again indicating a familiarity effect of prior passive exposure. Because the stimuli used for passive exposure and testing were the same two words but produced by two different groups of human speakers, this result shows that the familiarity effect for word categories transfers to new voices.

The response magnitude in NCM showed a larger absolute difference between word categories after passive exposure despite the variations in response patterns among individual recording sites and to different individual speakers. Thus, passive exposure led to an increase in the response difference between word categories. In addition, differences in response temporal profiles between words and between all stimuli increased over repetitions and were higher for the EXPOSED group. Thus, passive exposure increased the dissimilarity in neural responses to different stimuli^17^ and to different word categories. We further applied demixed principal component analysis, a dimensionality reduction approach, to decompose population activity into components that correspond to distinct stimulus parameters (word categories, individual speakers, and word-speaker combinations). Importantly, the component that captured the variance explained by word categories was higher for the EXPOSED group than for the NOVEL group and showed an increasing trend over stimulus repetitions in the NOVEL group. This suggests that the dynamic change in population response over passive exposure subserves an improved representation for word categories. Moreover, the decoding accuracy shows an increasing trend over stimulus repetitions for just the subgroups of sites with middle-range discrimination (top 25–50% decoding accuracy). This suggests that a subgroup of sites contributes most to the dynamic change in population representation during passive exposure.

Our results suggest that response adaptation during passive exposure may contribute to the formation of acoustic categories. This is consistent with observations in other systems showing that repeated exposure to sets of acoustic stimuli lead to response adaptation^24,25^ and can improve discrimination through statistical learning processes^26–28^. The word-specific increase in decoding accuracy that we observe indicates that the essential prototypic features that distinguish the two Dutch words, “*wit*” and “*wet*” (easily discriminated by fluent Dutch listeners) can be discriminated by neurons in zebra finch NCM, despite the surface differences between the voices of speakers and the two sexes. In addition, neural discrimination transfers to a new set of speakers, consistent with the behavioral study by Ohms et al.^10^. While we do not know how the prototypes become encoded, we do know that the prototypes are shared with all the exemplars of each of the two words, and thus are present on every stimulus and every trial. This repetition induces the known adaptation process in NCM^15^ (cf. Figure 3) and may do so more strongly for the repeated prototypical structure than for the surface features. Thus, we propose that adaptation is a candidate for a general processing mechanism across species that can serve speaker-independent coding and, more generally, the formation of acoustic categories for any type of stimulus.

Our findings are also consistent with the idea that the process of neural adaptation is not simply a uniform reduction in response magnitude, but instead could represent the functional reorganization of receptive field structures in secondary cortex to support improved detection of salient acoustic features that identify sound categories. While suggestive, the approach used here has not provided a strong test of whether adaptive changes in neural activity are a sufficient mechanism to underlie improvements in category perception; the speech stimuli used in the experiments vary along multiple dimensions. We anticipate that future work using synthetic stimuli that vary parametrically along a controlled acoustic dimension will provide a direct test. Studying dynamic changes in neural coding has the potential to reveal the mechanisms of neural plasticity that underlie the effects of sensory experience on stimulus discrimination and categorization. Enhanced categorization of salient signals may in turn subserve adaptive behavioral responses.

## Methods

### Subjects

Thirty-seven naïve adult zebra finches (*Taeniopygia guttata*) were used in this study (19 male, 18 female). One group received passive exposure (EXPOSED group: 8 male, 8 female) and the other did not receive prior exposure (NOVEL group: 11 male, 10 female). All birds lived in same-sex cages with ad libitum food and water in a general aviary maintained on a 12:12 light: dark cycle. All experimental protocols were approved by the Rutgers University Institutional Animal Care and Use Committee (IACUC). All methods were carried out in accordance with relevant guidelines and regulations. All methods are reported in accordance with ARRIVE guidelines.

### Surgeries

Birds were prepared for experiments by performing a craniotomy and implanting a metal pin on the skull. Birds were anesthetized with isoflurane (2–3% in oxygen), and bupivacaine was injected under the scalp to provide local analgesia. Birds were then placed in a stereotaxic apparatus. The scalp was incised at the midline and the skull was exposed. The skull was removed over the region of interest and a sealed chamber was formed over the recording area; a metal pin was attached to the skull anterior to this opening with dental cement to fix the bird’s head position during subsequent electrophysiological recordings Birds were injected with meloxicam (0.1–0.5 mg/kg) immediately after the surgery and were allowed to recover for at least 48 h before electrophysiological recordings.

### Electrophysiological recordings

Electrophysiological recordings were conducted on awake, restrained birds in a sound-attenuating chamber. Silicon probes with 4 shafts (NeuroNexus Technologies, Inc., Ann Arbor, MI) were used for recordings. Each probe included 16 recording sites (0.15–0.20 MΩ impedance at 1 kHz) arranged as a 4-by-4 grid with 200 μm between sites. Probes were prepared by being dipped into a DiI solution (0.1 mg/mL in ethanol) and allowed to dry in order to provide a fluorescent label of the insertion tracks for subsequent histological analyses. The body of the bird was comfortably restrained in a tube, and the metal pin was clamped to the stereotaxic apparatus. The craniotomy window was opened, and probes were then lowered to the surface of the brain above the NCM, one probe in each hemisphere. The four shanks were arranged in a rostral-caudal plane to target NCM based on stereotaxic coordinates (placement ranged from 0 to 0.5 mm rostral, 0.5–1 mm lateral of the bifurcation of the midsagittal sinus). Then the probes were slowly advanced with oil hydraulic micromanipulators (Narishige International USA, Inc., Amityville, NY) until bursting activity characteristic of NCM was observed. Continuous signals were bandpass filtered between 0.3 and 5 kHz, amplified 10,000 times, digitized at a sampling rate of 25 kHz, and saved to disk using Spike2 software (Cambridge Electronic Design, Cambridge, UK).

### Stimulus presentation

The stimuli were two naturally spoken Dutch words originally used in a behavioral study by Ohms et al.^10^ and generously shared with our laboratory. These Dutch words (“*wit*” and “*wet*”) were recorded from 20 native speakers (10 females, 10 males) at a 16-bit resolution with 44.1 kHz sampling rate. In the current study, the amplitude of all female and male stimuli was normalized by equalizing the root mean square of the average acoustic energy. During the experiment, all stimuli were played back at 65 dB SPL(A). Example spectrograms of the stimuli are shown in Fig. 1a. The initial and final consonants are the same for the two words, while the middle vowels differ.

For one group of birds (EXPOSED group), the speech stimuli were presented in two consecutive sessions. In the first (passive exposure) session, the two words produced by 10 human speakers (5 females and 5 males) were presented repeatedly, one word after the other. One word produced by 10 different speakers was presented 20 times (200 trials) in a pseudorandom order. Specifically, the 200 trials consisted of 20 consecutive blocks, each block consisted of the word produced by 10 different speakers (10 different stimuli). Therefore, within each block of 10 stimuli, any stimulus was only presented once; throughout the 200 trials, each stimulus was repeated 20 times in the 20 blocks. Then 20 repetitions of the other word produced by the same 10 speakers were presented (another 200 trials) in the same way, as described above. The order of the two words were randomized across birds. This passive exposure session was immediately followed by a testing session. In the testing session, the same two words produced by 10 different human speakers (5 females and 5 males) were presented 10 times each in a pseudorandom order with both words and speakers shuffled (200 trials). Specifically, the 200 trials consisted of 10 consecutive blocks, each block consisted of the 2 words produced by 10 different speakers (20 different stimuli). Therefore, within each block of 20 stimuli, any stimulus was only presented once; throughout the 200 trials, each stimulus was repeated 10 times in the 10 blocks. The other group of birds (NOVEL group) did not hear the passive exposure stimuli. Both the NOVEL and EXPOSED groups heard the same testing stimuli in the same format. In both sessions, stimuli were presented at an onset-onset inter-stimulus interval (ISI) of 6 s. The stimulus presentation order is shown in Fig. 1b.

### Histology

After the electrophysiological recordings, birds were deeply anesthetized with an overdose of pentobarbital (0.15 mL of 39 mg/mL), transcardially perfused with saline (0.9%) followed by paraformaldehyde (4% in phosphate-buffered saline), and decapitated. The brain was extracted and post-fixed with paraformaldehyde for at least 4 days. Then the brain was cut into 75-μm sagittal sections on a vibratome. Each unstained section was imaged under a fluorescence microscope with 450–490/515-cut-on and 510–560/590-cut-on nm excitation/emission filters for anatomical markers (via auto-fluorescence) and DiI tracks respectively. The two images from the same section were superimposed to create multicolor composite images for validating the recording sites. An example image of recording probes in NCM is shown in Fig. 1d.

### Data processing

Offline data analysis was performed with Spike2 and MATLAB scripts. Multi-unit spiking activity at each recording site was defined as any activity greater than 3 standard deviations from the mean amplitude of the raw signal across each recording session (Fig. 1e). Raw neural recordings were visually validated and recording channels with no signal or movement drifts across an entire recording session were excluded.

### Response magnitude and difference

As a metric of the overall neuronal spiking activity evoked by each stimulus in each NCM location, the multi-unit firing rates (FR) during each stimulus period (400 ms from stimulus onset; selected to coincide with the duration of the shortest word stimuli) were calculated for all trials for each recording site. For subsequent analyses, the multi-unit firing rates during the entire 400 ms duration were binned into 10-ms time windows and smoothed with a 5-point Gaussian window. To account for the variability in firing rates among all recording sites, the raw firing rate for each trial was divided by the mean firing rates during the 400 ms of stimulus presentation for all trials at each site to produce a percent of the mean response to all stimuli at that site (Relative Response Magnitude, RRM).

To quantify the neural discrimination of the two different word stimuli despite the variations in individual speakers, the absolute difference in RRM during stimulus presentation to the two words was calculated for each recording site:

$${\text{Absolute}}\;{\text{difference}}\;{\text{in}}\;{\text{RRM = }}{{\left| {{\text{RRM}}_{{{\text{Word1}}}} - {\text{RRM}}_{{{\text{Word2}}}} } \right|} \mathord{\left/ {\vphantom {{\left| {{\text{RRM}}_{{{\text{Word1}}}} - {\text{RRM}}_{{{\text{Word2}}}} } \right|} {\left( {{{\left( {{\text{RRM}}_{{{\text{Word1}}}} + {\text{RRM}}_{{{\text{Word2}}}} } \right)} \mathord{\left/ {\vphantom {{\left( {{\text{RRM}}_{{{\text{Word1}}}} + {\text{RRM}}_{{{\text{Word2}}}} } \right)} {\text{2}}}} \right. \kern-\nulldelimiterspace} {\text{2}}}} \right)}}} \right. \kern-\nulldelimiterspace} {\left( {{{\left( {{\text{RRM}}_{{{\text{Word1}}}} + {\text{RRM}}_{{{\text{Word2}}}} } \right)} \mathord{\left/ {\vphantom {{\left( {{\text{RRM}}_{{{\text{Word1}}}} + {\text{RRM}}_{{{\text{Word2}}}} } \right)} {\text{2}}}} \right. \kern-\nulldelimiterspace} {\text{2}}}} \right)}}$$

The absolute difference in response between words was compared between the two groups of birds both for the entire 400 ms stimulus duration and for a time period limited to just the vowel portion of words (mean window 100–200 ms period from stimulus onset).

### Adaptation rate

In previous studies in the laboratory, linear regression analyses between stimulus repetition and response magnitude have been performed to measure the adaptation rate of neural responses to repeated acoustic stimuli for each single unit or multi-unit recording site. The adaptation rate provides a measure of familiarity to acoustic stimuli: novel stimuli elicit higher initial responses which adapt rapidly, with higher negative slopes^16,17^. In the current study, different stimuli were presented in an intermixed order, therefore multilevel models were used to evaluate the adaptation rate where repetitions were nested within stimuli. The model assessed the change of response magnitude over stimulus repetitions for each recording site respectively. The repetition number of each stimulus was the predictor variable, and the RRM to each stimulus was the outcome variable. Fixed slopes were used for the predictor. Only the first 100 trials (5 repetitions of each stimulus) were included for each site when calculating the adaptation rates. The slope for each recording site was used as a measure of adaptation rate for that site. The models were analyzed using the lme4 package in R. The adaptation rates for the two groups were compared using Mann–Whitney U tests with an alpha level of 0.05.

### Neural distance

The dissimilarities between the neural responses were quantified using the cosine distance between the temporal profiles of neural responses to different stimuli. For each recording site, a 40-element vector was composed from the RRM computed for each 10-ms window across the 400-ms stimulus presentation for each trial; thus this vector represented the response temporal profile for that trial. The neural distance between pairs of response profiles was calculated as the cosine distance of any pair of such vectors, as illustrated in Supplementary Fig. S3. For any two *n*-dimensional vectors, ***A*** and ***B***, the cosine distance is calculated as

$$Cosine\:distance=1-\mathrm{cos}\left(\theta \right)=1-\frac{{\varvec{A}}\cdot {\varvec{B}}}{\Vert {\varvec{A}}\Vert \Vert {\varvec{B}}\Vert }=1-\frac{\sum_{i=1}^{n}{A}_{i}{B}_{i}}{\sqrt{\sum_{i=1}^{n}{A}_{i}^{2}}\sqrt{\sum_{i=1}^{n}{B}_{i}^{2}}}$$

where *A*_*i*_ and *B*_*i*_ are the *i*th components of vectors ***A*** and ***B*** respectively. Within each trial block consisting of the 20 different stimuli (20 trials), the cosine distance of neural responses was calculated for all stimulus pairs (i.e., 190 total pairs) and averaged to compute the neural distance for that trial block (“overall distance”). The neural distance was then calculated for each of the 10 trial blocks. In addition, to measure the neural dissimilarity between the two words, the cosine distance between responses to both words produced by the same speaker (10 total pairs) was calculated with the same methods for each trial block (“within-speaker distance”).

To evaluate the changes in neural distance across stimulus repetitions, a linear regression model between the cosine distance and trial block numbers was applied for each individual site. The calculated regression slopes were compared with 0 using one-sample Wilcoxon tests to determine if there was a change in cosine distance over stimulus repetitions. The mean value of cosine distance across all trial blocks was also calculated for each site. Then the mean distance and slopes of the linear regression were compared between groups to evaluate the effect of passive exposure on neural distance using Mann–Whitney U tests.

### Demixed principal component analysis

To separate and quantify the representation of word and speaker components in the NCM neuron populations, demixed principal component analysis (dPCA) was applied to the data. In brief, dPCA decomposes population activity into components that capture the variance of the data and demixes the dependencies of the population activity on experiment parameters^18^. Because the neural responses were recorded in different birds, to implement this approach, pseudo-populations were created to substitute for simultaneous recordings for each group of birds. The responses of N multi-unit sites were selected from the pool of all available recording sites to create a pseudo-population. With each recording site defining one response dimension, the activity of N multi-unit recording sites was represented in an N-dimensional neural space. The multi-unit firing rates during the stimulus presentation period were normalized by the mean firing rate across all stimuli and all trials, binned into 10 ms time windows and smoothed with a 5-point Gaussian window. The activity of neuron populations was decomposed into four distinct categories: condition-independent, word identity (“*wit*” vs. “*wet*”), individual speakers (N = 10), and a combination of word and speaker (each individual stimulus, N = 20).

To identify the contribution of each component to the population response changes during passive exposure, the dPCA method was applied using neural response data from consecutive trial blocks across stimulus repetitions. The variance explained by the first principal component in each category was calculated separately for each set of 60 trials/3 trial blocks (with a 20-trial/1-block step between adjacent data points) across the entire testing session (200 trials/10 blocks). Pseudo-populations of NCM neurons were created from the NCM recording sites for the two groups of birds (NOVEL and EXPOSED) respectively. To account for the difference in sample sizes between groups, samples were matched by randomly selecting from the group with more sites the same number of sites recorded for the smaller group. In this case, 213 multi-unit sites were randomly selected from the NOVEL group, and all 213 sites were used for the EXPOSED group to create the pseudo-populations. The variance explained by each dPCA component was evaluated across trial blocks and between groups. Linear regression models between variance explained by a component and trial block numbers were used to evaluate the trend of change over stimulus repetitions. The variance explained by each component was compared between groups to evaluate the effect of passive exposure using Mann–Whitney U tests.

### Neural decoding

To test the generalization of neural representation after passive exposure, decoding-based approaches (e.g. support vector machine methods, SVM) were applied to the population of neural responses recorded in the testing sessions. Pseudo-populations were created to substitute for simultaneous recordings for each group of birds. To create a pseudo-population, N multi-unit sites were randomly selected from the pool of all available recording sites. The model was trained to classify word identities based on the population responses to the word stimuli during one or more specific time bins or one time window (discussed below). To demonstrate that the representation of word categories can be generalized across individual human speakers, different combinations of speaker sets were used for training and validating the decoding algorithm. Specifically, a leave-one-out cross-validation procedure was repeated 10 times. Each time, population responses to the two word stimuli as produced by 9 of the 10 speakers were used as the training data to classify word identity, then the algorithm was used to predict the word identity for responses to the word stimuli as produced by the other 1 speaker. The accuracy for prediction was then averaged across the 10 different training/validation speaker sets. To account for the variations among different recording sites, the whole procedure above was repeated 100 times (100 iterations) with different combinations of sites to give a bootstrap-like estimate of the classification accuracy. These procedures were similar to the population decoding approach in Meyers et al.^29^. If the decoding model for word identity can classify words produced by novel speakers above chance rates, then it could indicate that the neural responses form a generalized representation of word identity across individual variants.

To evaluate the temporal dynamics of the decoding, the decoder was trained on the population response for each 10-ms time bin from 0 to 400 ms after stimulus onset, respectively, using the first 100 trials. For each time bin, 30 recording sites were randomly selected. The firing rates of the 30 recording sites during the 10-ms time period for the 100 trials were concatenated together. Among the 100 trials, a leave-one-out cross-validation procedure was repeated 10 times. Each time, responses to the words produced by different speakers were used to train and validate the classifier on word identity, as described above. The whole procedure was repeated for 100 iterations for each time bin and then repeated for all 40 time bins.

To evaluate the changes of population decoding over stimulus presentation, the decoder was trained on every 40 trials (2 trial blocks, with a 20-trial/1-block step). For each model, N recording sites were randomly selected from the recording sites for both groups. The firing rates of the 100 recording sites during the 100–200 ms since stimulus onset were divided into 10 time bins to create N × 10 features for training and validating the model. The 100–200 ms time window of the response was used because it is aligned with the vowel portion of the word stimuli. Among the 40 trials, responses to the words produced by different speakers were used to train and validate the classifier on word identity, in a leave-one-out cross-validation procedure as described above. The whole procedure was repeated 100 times for every 40 trials over trial blocks.

To evaluate the decoding accuracy as a function of the numbers of recording sites, different numbers of sites (N = 1, 5, 10, 15, 20, 30, 50, 75, 100, 125, 150, 200) were randomly selected from the multi-unit recording sites of NOVEL group and the EXPOSED group respectively. The entire decoding approach was applied for 1000 iterations, and the decoding accuracy was averaged across all trial blocks. Similarly, to evaluate the decoding performance for individual sites, the model was also applied to each multi-unit recording site respectively, and the averaged decoding accuracy across trial blocks were used as an estimate of decoding accuracy for the individual site.

To evaluate the contribution of individual NCM sites, all recording sites of each group were further divided into four quartiles based on their individual decoding accuracy, with one quarter of all sites in each subgroup. Then the decoding approach above was applied to each quartile respectively, except that random selections of 20 sites were used for all subgroups due to the relatively smaller numbers of sites in the quartiles.

To determine significance, chance performance was calculated by training the decoder with the same data but with shuffled identifiers of word identity. After the decoding accuracy was calculated, Wilcoxon signed-rank tests were performed for each group or time bin between the testing data and shuffle control data using Bonferroni adjusted alpha levels. In addition, the difference in decoding accuracy between individual sites of the two groups of birds was compared with Mann–Whitney U test. Linear regression models between the decoding accuracy and trial block numbers were used to evaluate the trend of change over stimulus repetitions.

## Supplementary Information

Supplementary Information.

## Data Availability

The datasets generated during and analyzed during the current study are available from the corresponding author on reasonable request.
