# Supplementary material for: Dynamic encoding of phonetic categories in zebra finch auditory forebrain
Source: Sci Rep. 2023 Jul 10;13:11172. doi: 10.1038/s41598-023-37982-5 (PMC10333302; doi:10.1038/s41598-023-37982-5)
Supplement: Supplementary file 1 — Supplementary Information. [file 41598_2023_37982_MOESM1_ESM.pdf]

Supplementary Information

Dynamic encoding of phonetic categories in zebra finch auditory forebrain

Wanyi Liu[1, *] & David S. Vicario[1, *]

1. Department of Psychology, Rutgers, the State University of New Jersey, Piscataway, NJ 08854, United States

* Wanyi Liu: wl535@scarletmail.rutgers.edu

* David S. Vicario: vicario@psych.rutgers.edu

Supplementary Methods and Data

Neural response and adaptation during the passive exposure session

Neural responses to the word stimuli during the initial passive exposure session were recorded from multi-unit sites in NCM of the EXPOSED group of birds. The extraction of multi-unit activity and the normalization of neural responses across recording sites were the same as for recordings made during the testing sessions (see Methods). During the passive exposure session, the two Dutch words (*"wet"* and *"wit"*) produced by 10 different human speakers were presented repeatedly. On Trials 1-200, one word (Word 1) produced by 10 speakers was presented (20 repeats of each of the 10 stimuli). On Trials 201-400, the other word (Word 2) produced by the **same** 10 speakers was similarly presented (see Methods and **Fig. 1b**). The order of the two words was randomized between birds. Preliminary analyses did not show differences related to word order. Therefore, the data from different presentation orders were combined. The average relative response magnitude (RRM) of 192 multi-unit NCM sites to each trial in the passive exposure session are shown in **Fig. S1**. Responses decreased over stimulus repetitions for Word 1, then increased when Word 2 was introduced, then decreased again.

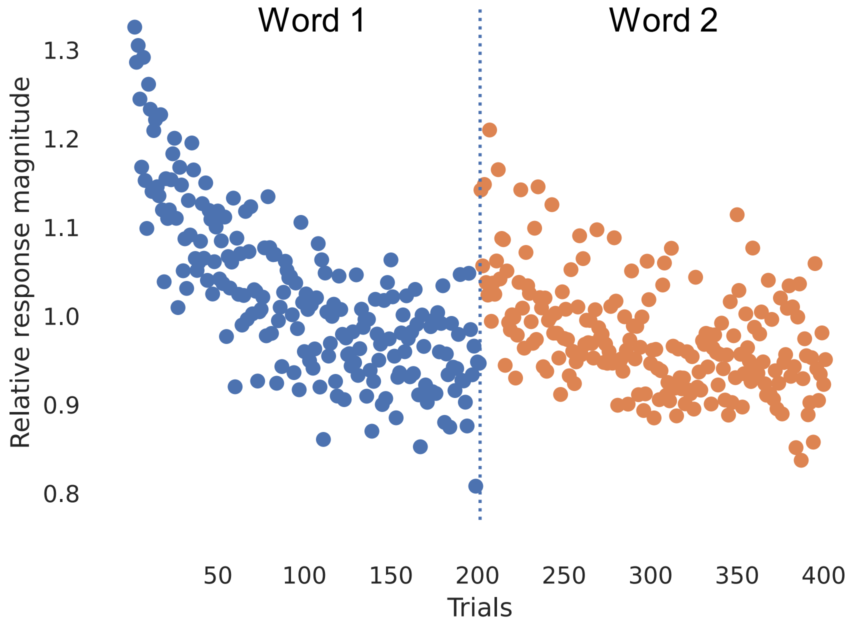

Figure S1. Average relative response magnitude (RRM) to the word stimuli during the initial passive exposure session for 192 NCM recording sites in the EXPOSED group. During the passive exposure session, the two Dutch words (*"wet"* and *"wit"*) produced by 10 different human speakers were presented repeatedly. On Trials 1-200, Word 1 (blue) produced by 10 speakers was presented for 20 repetitions each. On Trials 201-400, Word 2 (orange) produced by the **same** 10 speakers was presented (see Methods and Fig. 1b). Each dot represents the average RRM across all recording sites for each trial during stimulus presentation (0-400 ms since stimulus onset).

To quantify these transition effects, the multi-unit firing rates during stimulus presentation were compared (1) between the first 50 trials of each word (5 repetitions of each stimulus) and (2) between the last 50 trials of Word 1 (before word transition) and the first 50 trials of Word 2 (after word transition) (**Fig. S2a**). Neural responses during the first 50 trials of Word 1 were higher than those during the first 50 trials of Word 2 (Word 1: Mean = 184.83 Hz, SD = 55.80 Hz; Word 2: Mean = 167.71 Hz, SD = 52.37 Hz. Wilcoxon signed-rank test, $Z = 3256.5$, $p < 0.001$, $N = 192$ multi-unit sites). This suggests that exposure to the one word produced by a set of speakers caused a familiarity effect of the speakers' voices that could transfer to the other word spoken by the same speakers. However, neural responses during the first 50 trials of Word 2 were significantly higher than those during the last 50 trials of Word 1 (Word 1: Mean = 165.61 Hz, SD = 53.10 Hz; Word 2: Mean = 167.71 Hz, SD = 52.37 Hz. Wilcoxon signed-rank

test, $Z = 3920$, $p < 0.001$, $N = 192$). This suggests that presentation of a different word introduced novelty, despite the familiarity of the same speakers' voices.

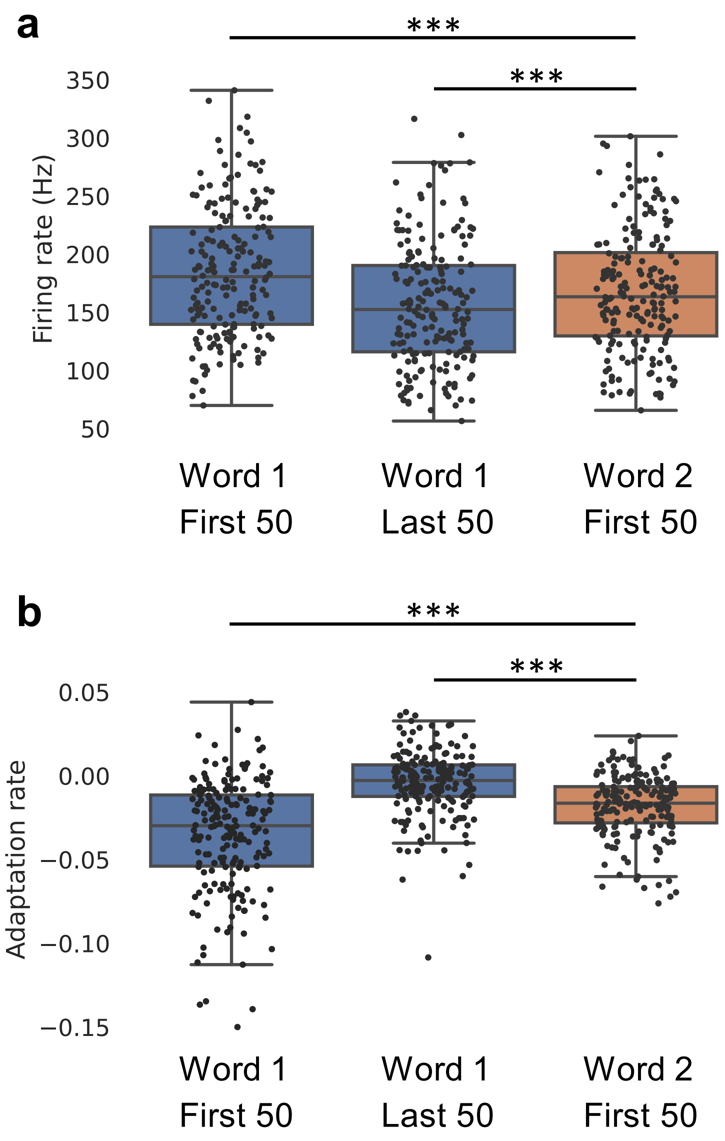

Figure S2. Firing rates (a) and adaptation rates(b) for each recording site in the passive exposure session. The multi-unit firing rates and adaptation rates were calculated in the same way as that for the testing stimuli (see Methods), except that the adaptation rate was calculated for each word and different trial blocks separately: across the first 50 trials of Word 1, the last 50 trials of Word 1 (before word transition), and the first 50 trials of Word 2 (after word transition).

To quantify the changes in neural response during exposure and evaluate the familiarity effect, the adaptation rates were also calculated separately for Word 1 and Word 2 for each site. For both words, the adaptation rates were significantly lower than zero during the first 50 trials

(Word 1: Mean = -0.036, SD = 0.035, one sample Wilcoxon signed-rank test: Z = 783, $p < 0.001$. Word 2: Mean = -0.018, SD = 0.019, one sample Wilcoxon signed-rank test: $Z = 1229$, $p < 0.001$), suggesting that neural responses showed a decrease over stimulus presentation. The adaptation rates were significantly "steeper" (more negative) during the first 50 trials of Word 1 than those for Word 2 (Wilcoxon signed-rank test, $Z = 4593$, $p < 0.001$, $N = 192$, **Fig. S2b**). This also suggests a familiarity effect to the speakers' voices after exposure to one word can transfer to the other word spoken by the same speakers. To evaluate whether the adaptation rate changed between the transition from Word 1 to Word 2, the adaptation rates were also calculated for the last 50 trials of Word 1 (before word transition), and then compared to the adaptation rates of the first 50 trials of Word 2 (after word transition). The adaptation rates were significantly "steeper" (more negative) for the first 50 trials of Word 2 (after word transition) than that of the last 50 trials of Word 1 (before word transition) (Wilcoxon signed-rank test, $Z = 4424$, $p < 0.001$, $N = 192$ multi-unit sites, **Fig. S2b**). The change in adaptation rate after word transition is consistent with the increase in firing rates, suggesting a novelty effect to the presentation of a different word despite the familiarity to the same speakers' voices.

Calculation of cosine distances between temporal response profiles

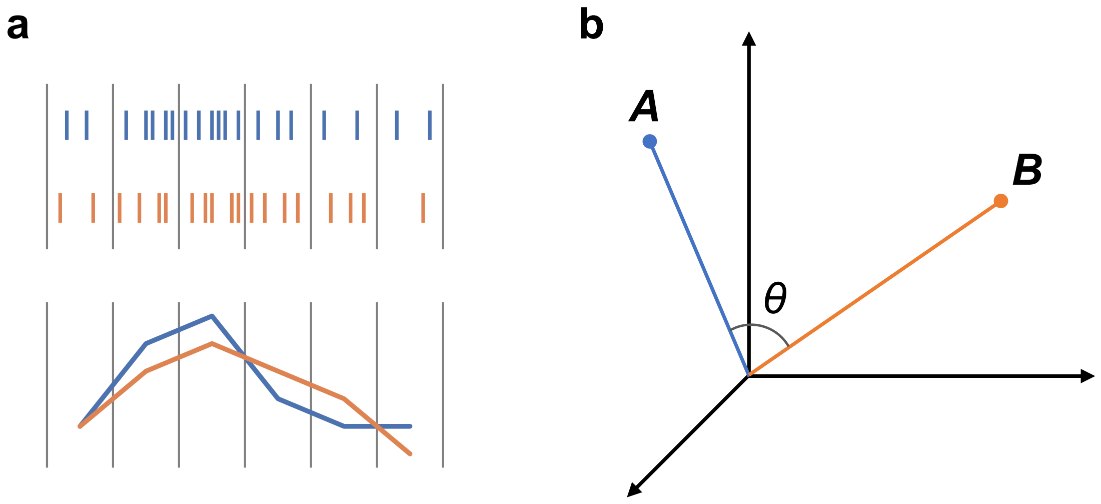

$$\boldsymbol{A} = [A_1, A_2, \ldots, A_n]$$

$$\boldsymbol{B} = [B_1, B_2, \ldots, B_n]$$

$$cos(\theta) = \frac{\boldsymbol{A} \cdot \boldsymbol{B}}{\|\boldsymbol{A}\| \|\boldsymbol{B}\|} = \frac{\sum_{i=1}^{n} A_i B_i}{\sqrt{\sum_{i=1}^{n} A_i^2} \sqrt{\sum_{i=1}^{n} B_i^2}}$$

$$cosine\ distance = 1 - \cos(\theta)$$

Figure S3. Illustration of cosine distance between response temporal profiles. Raw multi-unit activity was thresholded over the 400 ms response period (see Methods and Fig. 1e), Normalized by the mean firing rate of each recording site, then binned into 10ms time windows to form 40 dimensional vectors. (a) Two examples of the resulting vectors created from spiking activity and normalized response profiles. (b) Calculation of the cosine distance. The cosine distance between the two vectors was calculated as a measure of the neural distance between the two response profiles as shown.
